# Chemokine Oligomers and the Impact of Fondaparinux Binding

**DOI:** 10.1021/jasms.4c00142

**Published:** 2024-06-05

**Authors:** Gergo Peter Szekeres, Douglas P. Dyer, Rebecca L. Miller, Kevin Pagel

**Affiliations:** † Institute of Chemistry and Biochemistry, Freie Universität Berlin, 14195 Berlin, Germany; ‡ Fritz Haber Institute of the Max Planck Society, 14195 Berlin, Germany; § Wellcome Centre for Cell-Matrix Research, Manchester Academic Health Science Centre University of Manchester, M13 9PT Manchester, U.K.; ∥ Geoffrey Jefferson Brain Research Centre, Manchester Academic Health Science Centre, University of Manchester, M6 8FJ Manchester, U.K.; ⊥ Center for Glycomics, Department of Cellular and Molecular Medicine, University of Copenhagen, Blegdamsvej 3, DK-2200 Copenhagen, DK

## Abstract

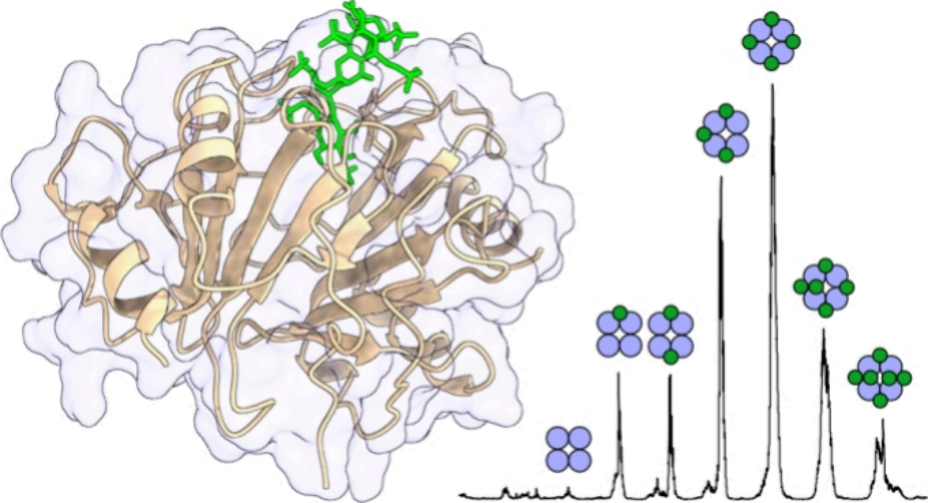

Heparin, a widely used clinical anticoagulant, is generally well-tolerated; however, approximately 1% of patients develop heparin-induced thrombocytopenia (HIT), a serious side effect. While efforts to understand the role of chemokines in HIT development are ongoing, certain aspects remain less studied, such as the stabilization of chemokine oligomers by heparin. Here, we conducted a combined ion mobility-native mass spectrometry study to investigate the stability of chemokine oligomers and their complexes with fondaparinux, a synthetic heparin analog. Collision-induced dissociation and unfolding experiments provided clarity on the specificity and relevance of chemokine oligomers and their fondaparinux complexes with varying stoichiometries, as well as the stabilizing effects of fondaparinux binding.

## Introduction

Glycosaminoglycans (GAGs) are linear polysaccharides with varying sulfation patterns that are omnipresent in tissues either in free form or as proteoglycans. Among GAGs, heparin stands out for its exceptionally high degree of sulfation, rendering it the most negatively charged biomolecule known. Heparin is used as a clinical anticoagulant, as it interacts with the coagulation cascade:^1,2^ it binds to antithrombin III,^3^ resulting in conformational changes in the molecule, which in turn activates antithrombin III.^1,4^ This activated complex can then bind to thrombin and factor Xa (as well as other proteases in the coagulation cascade), which eventually results in up to 1000-fold increase in inhibition of blood clotting.^5^ In the case of thrombin inactivation, the formation of the antithrombin III–heparin–thrombin ternary complex strongly relies on the electrostatic interactions with the highly negatively charged heparin molecule, requiring a chain length of at least 18 monosaccharide units.^6^ Therefore, the highest anticoagulant activity can be observed for the purified unfractionated heparin samples.

Unfortunately, while unfractionated heparin is very promising in acute coagulation-related cases, it can lead to heparin-induced thrombocytopenia (HIT) in ∼1% of patients, often evoking the exact opposite of the desired effects of this drug.^7^ HIT can be classified into two different cases.^8,9^ In HIT *type I*, heparin directly interacts with platelets, leading to their clumping. This condition can arise within a day from heparin administration, but usually does not lead to thrombotic effects. The platelet count returns to normal in a few days after heparin is withdrawn from the patient.^9^ In contrast, HIT *type II* is immune-system-mediated and has a high risk of leading to thrombosis. HIT *type II* (for simplicity, further referred to as HIT) is induced by the association of heparin with platelet factor 4 (PF4 or CXCL4), a small chemokine molecule, with which it forms large aggregates.^9,10^ These large complexes are antigens for the anti-PF4-heparin antibodies, most often immunoglobulin G (IgG), and the associated heparin-PF4-IgG complex then activates platelets. This leads to the emission of platelet microparticles and the elimination of activated platelets from the bloodstream by the spleen, resulting in the reduced platelet counts observed in HIT.^10,11^

Chemokines exhibit diverse structures and oligomeric states, with some existing primarily as monomers while others form homo-oligomers. For instance, CXCL4 can exist as monomers or homotetramers, while others like CCL2 may appear as monomers or homodimers, and CCL7 typically exists as a monomer. Despite abundant research on chemokine–GAG interactions, there remains a significant knowledge gap regarding the relevance and structural characteristics of these complexes as a result of the high complexity of chemokine functions^12−14^ and the structural diversity of GAGs.^15^

In this study, we employed a combined ion mobility-native mass spectrometry approach to investigate three different chemokines (CCL7, CCL2, and CXCL4) and their interactions with fondaparinux, a synthetic heparin analog. Our findings provide insights into the specificity and relevance of chemokine oligomers and suggest that the CCL2 and CXCL4 homodimers are nonfunctional species. We observed that both the nonfunctional dimers and the specific CXCL4 homotetramers are stabilized by fondaparinux binding. The tetramer stability increases with the number of fondaparinux molecules in the complex, suggesting a progressive reliance on their bridging around and intercalation into the tetramer.

## Materials and Methods

CCL2, CCL7, and CXCL4 chemokine samples were purchased from Protein Foundry LLC (USA). Each protein sample was dissolved in Milli-Q water (18.2 MΩ·cm) at 1 mg/mL concentration, and 10 μL aliquots were stored at −80 °C. On the day of the experiment, the protein aliquots were thawed, and 20 μM solutions were prepared with 250 mM ammonium acetate buffer (Sigma).

Fondaparinux (Sigma) was desalted on a HiTrap desalting column (Cytiva) by a Knauer FPLC at a 1 mL/min flow rate, and a 200 μM stock solution was prepared in Milli-Q water. 4 μL of the respective 20 μM chemokine solution was mixed with 0.4 μL of the fondaparinux stock solution to yield a 1:1 molar ratio solution for mass spectrometry studies.

Native ion mobility-mass spectrometry studies were performed on a Waters Synapt G2-S instrument modified with a drift tube ion mobility cell. The respective samples were loaded into Pd/Pt-coated borosilicate glass capillaries produced in-house for nanoelectrospray ionization. The relevant instrumental parameters were as follows: capillary voltage −1000 V, cone voltage −30 V, cone offset −15 V, trap gas flow −4 mL/min, and He pressure in mobility cell −1.800 Torr. To maintain the softest conditions and transmit all possible complex stoichiometries, mass spectra were recorded while bypassing ion mobility separation.

Collision-induced dissociation and collision-induced unfolding data were recorded in the 0–60 V collision voltage range with 10 V steps on samples with identical composition as for native mass spectrometry and ion mobility-mass spectrometry data. Except for the tandem-mass-spectrometry data, mass selection during the acquisition was not employed.

The ion mobility-mass spectrometry data were extracted from Waters MassLynx (if necessary, mean-smoothed within a 2-point window), and further processed in Origin Pro 2020, UniDec,^16^ and a custom-written Python script. Each data set comprises of the average signal of 50< single spectrum acquisitions, and for each sample, at least two data sets were recorded. The protein and protein–heparin complex structures were visualized with ChimeraX.^17^ Rotationally averaged absolute collision cross sections in He drift gas were calculated based on the Mason–Schamp equation as described previously.^18^

## Results and discussion

**Figure 1:**
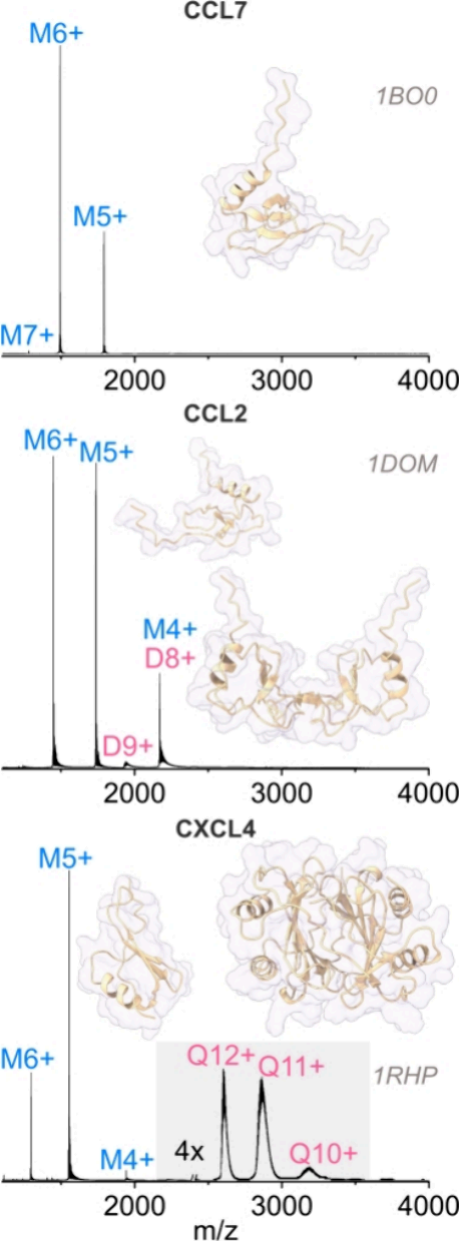
Native mass spectra of the chemokines CCL7, CCL2, and CXCL4 with the monomeric and oligomeric structures rendered from crystallographic data.^17,19−21^ The blue and pink numbers mark the mono- and oligomeric charge states, respectively. The intensity of the CXCL4 tetramers was boosted 4-fold for clarity.

Three distinct chemokines—CCL7, CCL2, and CXCL4—were selected based on their inherent tendencies for oligomerization. As illustrated in Figure 1, CCL7 predominantly exists as a monomer under native conditions, whereas CCL2 and CXCL4 are also observed in homodimeric and homotetrameric form, respectively. Notably, while CXCL4 readily forms tetramers, dimer formation is not observed under native conditions.

In the native mass spectrum of each chemokine, the 5+ and 6+ charge states of the monomers are present, serving as a reference for comparison. The 5+ charge state was chosen for further gas-phase structural characterization to minimize charge-induced structural alterations. Given their distinct tendencies for homo-oligomerization in the gas phase, these chemokines offer an ideal opportunity to investigate the impact of association with a heparin analog on their gas-phase complexes.

**Figure 2:**
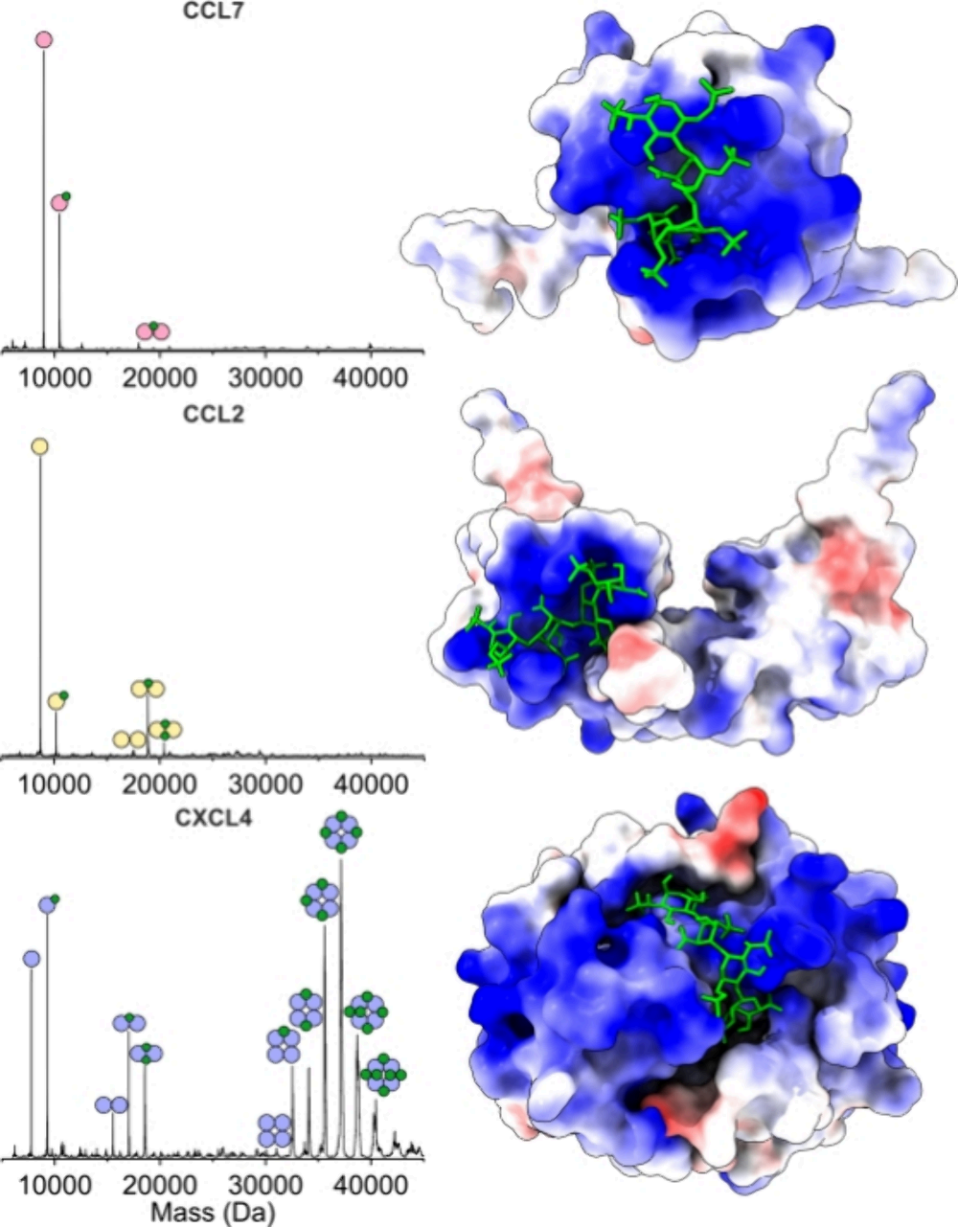
Deconvoluted native mass spectra of the 1:1 molar ratio mixture of fondaparinux with CCL7, CCL2, and CXCL4, respectively, with corresponding docking results at the highest oligomeric states with a heparin tetrasaccharide. All three chemokines appear as monomeric and dimeric structures with fondaparinux, and the majority of CXCL4 molecules form tetramers. The schematics assign the peaks to the different complexes where the small green circles mark fondaparinux, while the larger colored circles each mark a chemokine monomer. The docking results do not explain the higher extent of dimerization of CCL2. The deconvolution was performed in UniDec,^16^ and the docking experiments were executed by ClusPro.^22^

The mass spectra of the 1:1 solutions of chemokine:fondaparinux exhibit a wide range of species, necessitating spectral deconvolution for a clearer understanding of complex formation. Deconvoluted mass spectra of each chemokine:fondaparinux solution are depicted in Figure 2, with representative raw mass spectra provided in the Supporting Information (see Figure S1). In the deconvoluted mass spectrum of CCL7, the majority of molecules remain monomeric without forming complexes with fondaparinux, while a lower-intensity peak indicates the presence of a 1:1 complex. Intriguingly, a small fraction of molecules also forms a 2:1 CCL7:fondaparinux complex, suggesting that heparin association induces CCL7 dimerization to a limited extent under native conditions. Similarly, in the case of CCL2, the majority of monomers remain unbound with a minor portion forming a 1:1 complex with fondaparinux. Meanwhile, most CCL2 dimers interact with either one or two fondaparinux molecules. Comparing the monomer-to-dimer peak ratios in the mass spectrum of pure CCL2, it can be inferred that the majority of dimers result from interactions with fondaparinux rather than pre-existing CCL2 dimers engaging with fondaparinux molecules.

Docking simulations by ClusPro^22^ based on a heparin tetrasaccharide and the *1BO0*,^21^*1DOM*,^19^ and *1RHP*(20) structures show that each chemokine can interact with heparin analogs via an extended positive patch on their surface (Figure 2). However, in the case of the CCL2 dimer structure,^19^ all the stable complexes have the heparin molecule located at a highly positive patch on only one of the CCL2 monomer units (example shown in Figure 2 and Supporting Figure S2). This does not sufficiently support the experimental observations of increased dimerization; therefore, it is expected that more significant rearrangements occur in the CCL2 dimer structure upon association with fondaparinux,^23,24^ which are not predicted by docking experiments on rigid protein structures. Conclusions based solely on *in silico* docking simulations may therefore lead to a biased picture of heparin-chemokine interactions.

Incubation of CXCL4 with fondaparinux leads to a large number of complexes with distinct stoichiometries, as shown by the native mass spectrometric data (Figure 2 and Supporting Figure S2). As opposed to CCL2, most CXCL4 molecules do not retain a pure monomeric state. More than half of the monomers are in a 1:1 (rarely in 1:2) complex with fondaparinux. The majority of the CXCL4 molecules, however, form homotetramers in the presence of fondaparinux. Together with the formation of dimeric CXCL4 complexes that are only present marginally in the pure protein solution, these results show that fondaparinux tends to induce CXCL4 oligomerization. Most tetramers are present in a 4:4 complex with fondaparinux, but other complexes with up to six fondaparinux molecules are also found in significant quantities with the pure tetramer barely present (Figure 2, bottom).

In mass spectrometry, oligomeric complexes sometimes dissociate, because of unintended ion activation. To assess whether the observed CXCL4 dimers are the result of ion activation and subsequent, unusual symmetric dissociation of a tetramer rather than assembly in solution, collision-induced dissociation was performed on the 11+ 4:2 CXCL4:fondaparinux complex.

**Figure 3:**
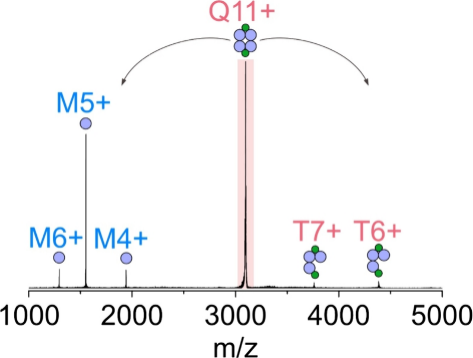
Collision-induced dissociation mass spectrum of the 11+ 4:2 CXCL4:fondaparinux complex at 30 V collision voltage. The parent ion signal is highlighted in red.

As shown in Figure 3, the 4:2 complex dissociates asymmetrically^25^ into the monomeric CXCL4 and the 3:2 CXCL4:fondaparinux complex at 30 V collision voltage. CXCL4 dimers were not observed in these experiments, which indicates that the dimerization of CXCL4 is induced by fondaparinux and is not an artifact of an unusual symmetric gas-phase disassembly of tetrameric complexes. Moreover, these experiments showcase the strong interaction between CXCL4 and fondaparinux, which is more resistant to ion activation than the interactions within the tetramer. This can result from the extended positive belt around the CXCL4 tetramer,^20,22^ allowing the fondaparinux to bridge over the structure. In addition, fondaparinux can intercalate into the tetrameric structure, which can further stabilize the complex from within (Figure 2 and Figure S3, Supporting Information).

Collision-induced unfolding experiments via ion mobility-mass spectrometry are often performed to understand the specificity and physiological relevance of protein oligomers observed in the gas phase.^26,27^ Here, the arrival time distribution from drift tube ion mobility measurements provides information on the relative size and shape of gas-phase ions, which can be converted into rotationally averaged collision cross sections based on the Mason–Schamp equation.^18^ Sequential collision-induced unfolding experiments consist of ion mobility spectrometry at increasing collision voltages, thus providing three-dimensional information related to the structural stability of the ions.

**Figure 4:**
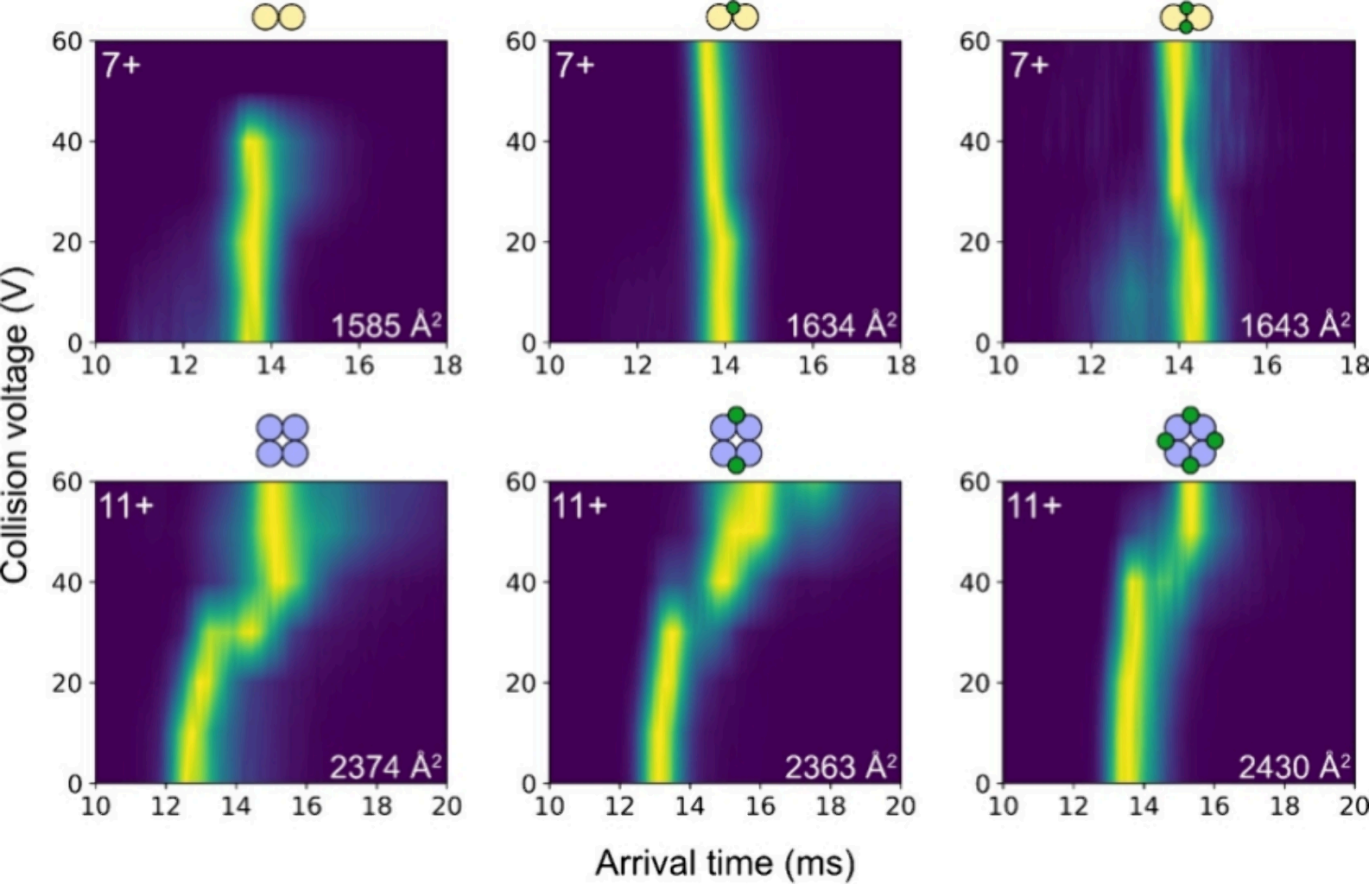
Collision-induced unfolding of the 7+ 2:0, 2:1, and 2:2 CCL2:fondaparinux complexes (top) and the 11+ 4:0, 4:2, and 4:4 CXCL4:fondaparinux complexes (bottom). The white numbers in the bottom right corner of the panels mark the rotationally averaged collision cross section in He drift gas at 0 V collision voltage.

The collision-induced unfolding experiments on the chemokine:fondaparinux 1:0 and 1:1 species show only slight changes over the entire voltage ramp (Figure S4, Supporting Information). The case of the CCL2 and CXCL4 dimers, however, is more complex. The pure dimers fully unfold and dissociate at 30–40 V, while fondaparinux keeps them at least partially intact across the entire collision voltage range (Figures 4 and S5, Supporting Information).

However, a close inspection of the unfolding profile across the voltage ramp shows that neither of the 7+ dimers follows the often-observed two-stage expansion, where one of the monomer units is unfolded first to counteract the increased energy, followed by the complete unfolding of the protein dimer.^28^ While the 8+ CCL2 dimer does show resemblance of a two-stage expansion profile (Figure S6, Supporting Information), the observations made on the 7+ species together with the fact that their abundance is marginal in the mass spectra of pure protein solutions suggest that the CCL2 and CXCL4 dimers are nonfunctional species. This is in agreement with previous findings, where CCL2 monomers were sufficient for receptor activation,^29,30^ and the suggested role of possible oligomerization was simply to increase the local concentration of chemokines.^31^

The effect of fondaparinux on the integrity of oligomers is well-represented in both the collision cross sections and the collision-induced unfolding of the CXCL4 tetramer complexed with 0–4 fondaparinux molecules (Figures 4 and S7, Supporting Information). While the collision cross-section of the nonfunctional CCL2 dimer increases by 3% upon interaction with a single fondaparinux molecule, the CXCL4 tetramer undergoes compaction when binding 1–2 fondaparinux molecules (Figures 4 and S7, Supporting Information). Without fondaparinux, the tetramer starts unfolding at 30 V, followed by further unfolding at 40 V and slight compacting subsequently. This is in agreement with the observations in true solution-phase protein oligomers.^28^ With only one fondaparinux, these tendencies change: the first stage of unfolding takes place at 40 V, and at higher voltages, continuous expansion is observed (Figure S7, Supporting Information). This is similar to the tetramers with 2–4 fondaparinux molecules, but with increasing number of fondaparinux, the unfolding begins at increasingly higher voltages and becomes much less pronounced (Figures 4 and S7, Supporting Information). As collision with the drift gas leads to the slight activation of ions in the ion mobility cell, the CXCL4 tetramers with 4< fondaparinux molecules are not observed in these experiments (see Figure S8, Supporting Information); however, the results conclusively demonstrate the stabilizing effect of fondaparinux on chemokine oligomers.

Beyond the expected dissociation behavior of higher oligomers, fragmentation trends that are characteristic of glycosaminoglycans (GAGs) can also be observed. The most significant fragmentation channel of sulfated GAGs in collision-induced dissociation is the neutral loss of sulfates. This phenomenon is also observed in 1:1 chemokine-fondaparinux complexes. Here the first sulfate loss occurs at 40, 50, and 60 V for CXCL4, CCL2, and CCL7, respectively (Figure S9, Supporting Information), surprisingly, however, without dissociation of the fondaparinux from the complex. This highlights the strong electrostatic nature of the interaction between the chemokines and fondaparinux.

## Conclusions

In this work, an ion mobility-native mass spectrometry study was performed on chemokine-fondaparinux complexes to gain a more in-depth understanding of their structural properties, especially the stabilizing forces within. Three chemokines—CCL7, CCL2, and CXCL4—were chosen as model proteins due to their different oligomerization tendencies in solution. The results from native mass spectrometry show that fondaparinux, a synthetic heparin analog, induces oligomerization, and CXCL4 dimers can also be observed in its presence, which are not prominent species in the pure protein solution. However, the mass spectrometric and collision-induced unfolding results indicate that the CCL2 and CXCL4 dimeric species are nonfunctional dimers, which questions their relevance in solution. The tendency of fondaparinux binding and oligomerization increased in the order of CCL7, CCL2, and CXCL4, with the majority of CXCL4 molecules binding at least one fondaparinux molecule and forming tetramers. The results show that fondaparinux binding can stabilize the complexes. This is the case specifically for the true CXCL4 tetramer, where the bridging of fondaparinux molecules across the extended positive belt around the tetramer and their intercalation into the structure can both contribute to the stability of the complexes. Moreover, the sulfate loss patterns in the collision-induced dissociation of the 1:1 chemokine:fondaparinux structures highlight the electrostatic nature of the binding.

## Supporting Information Available

Representative raw native mass spectra for Figure 2; example structure for the CCL2 dimer:heparin tetrasaccharide complex; example structures for the CXCL4 tetramer:heparin tetrasaccharide complex; collision-induced unfolding of chemokine monomers with and without fondaparinux; collision-induced unfolding and dissociation of CCL2 and CXCL4 dimers with and without fondaparinux; collision-induced unfolding of the CXCL4 tetramer with and without fondaparinux; collision-induced dissociation mass spectrum of the 11+ 4:4 CXCL4:fondaparinux complex; collision-induced sulfate loss of chemokine monomer:fondaparinux complexes (PDF)
