# Supplementary material for: Chemokine Oligomers and the Impact of Fondaparinux Binding
Source: J Am Soc Mass Spectrom. 2024 Jun 5;35(7):1550–5. doi: 10.1021/jasms.4c00142 (PMC11228995; doi:10.1021/jasms.4c00142)
Supplement: Supplementary file 1 — js4c00142_si_001.pdf [file js4c00142_si_001.pdf]

Supporting Information

Chemokine Oligomers and the Impact of Fondaparinux Binding

Gergo Peter Szekeres,[a,b] Douglas P. Dyer,[c,d] Rebecca L. Miller,[e] Kevin Pagel[a,b,*]

a Institute of Chemistry and Biochemistry, Freie Universität Berlin, 14195 Berlin, Germany

b Fritz Haber Institute of the Max Planck Society, 14195 Berlin, Germany

c Wellcome Centre for Cell-Matrix Research, Manchester Academic Health Science Centre University of Manchester, M13 9PT Manchester, United Kingdom

d Geoffrey Jefferson Brain Research Centre, Manchester Academic Health Science Centre, University of Manchester, M6 8FJ Manchester, United Kingdom

e Center for Glycomics, Department of Cellular and Molecular Medicine, University of Copenhagen, Blegdamsvej 3, DK-2200 Copenhagen, Denmark

*E-mail: kevin.pagel@fu-berlin.de

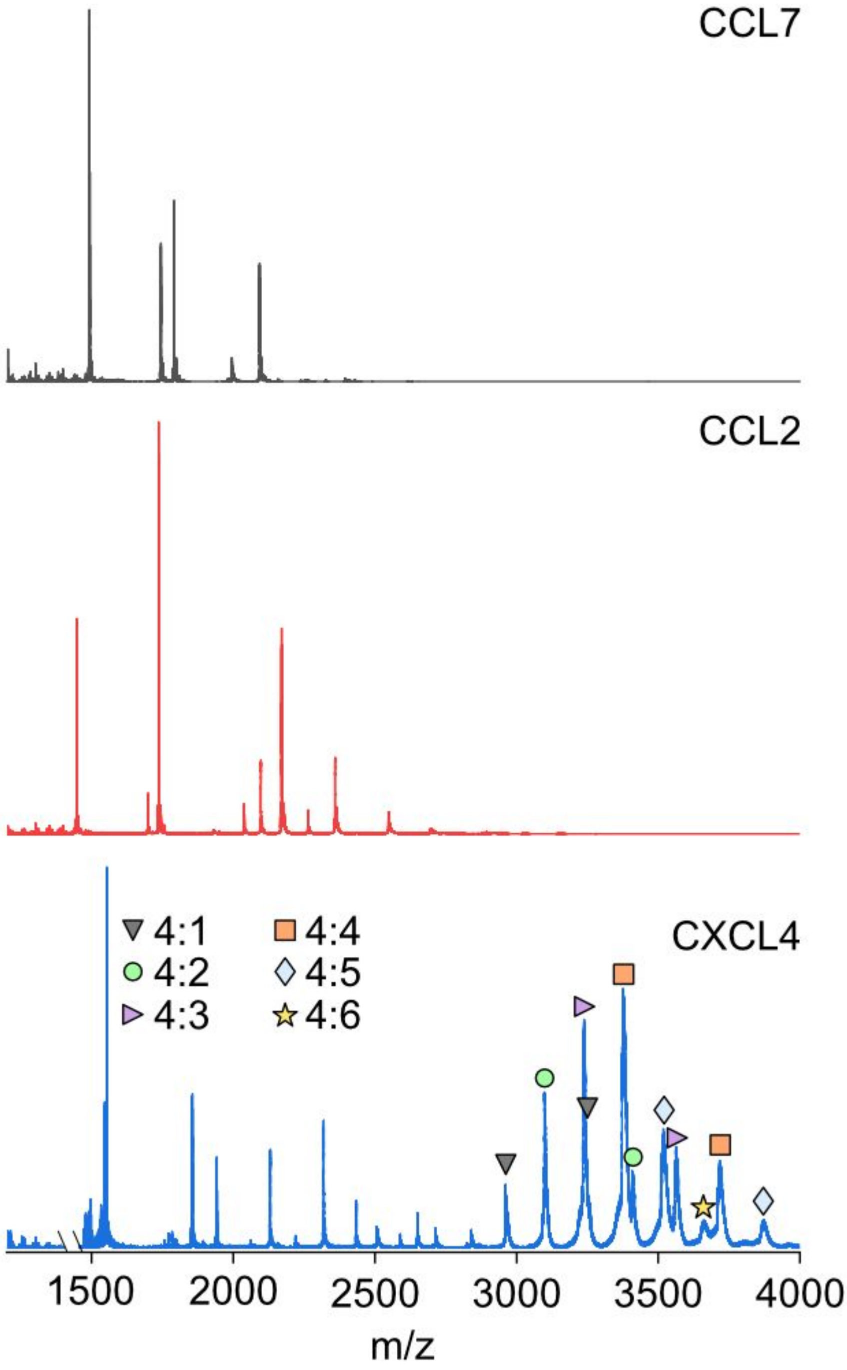

Figure S1. Representative native mass spectra of 20 μM CCL7, CCL2, and CXCL4 solutions with 20 μM fondaparinux (black, red, and blue traces, respectively). The intensity of the lower mass region in the CXCL4 trace was reduced to emphasize the relevant spectral range, and the different stoichiometry species were annotated for clarity.

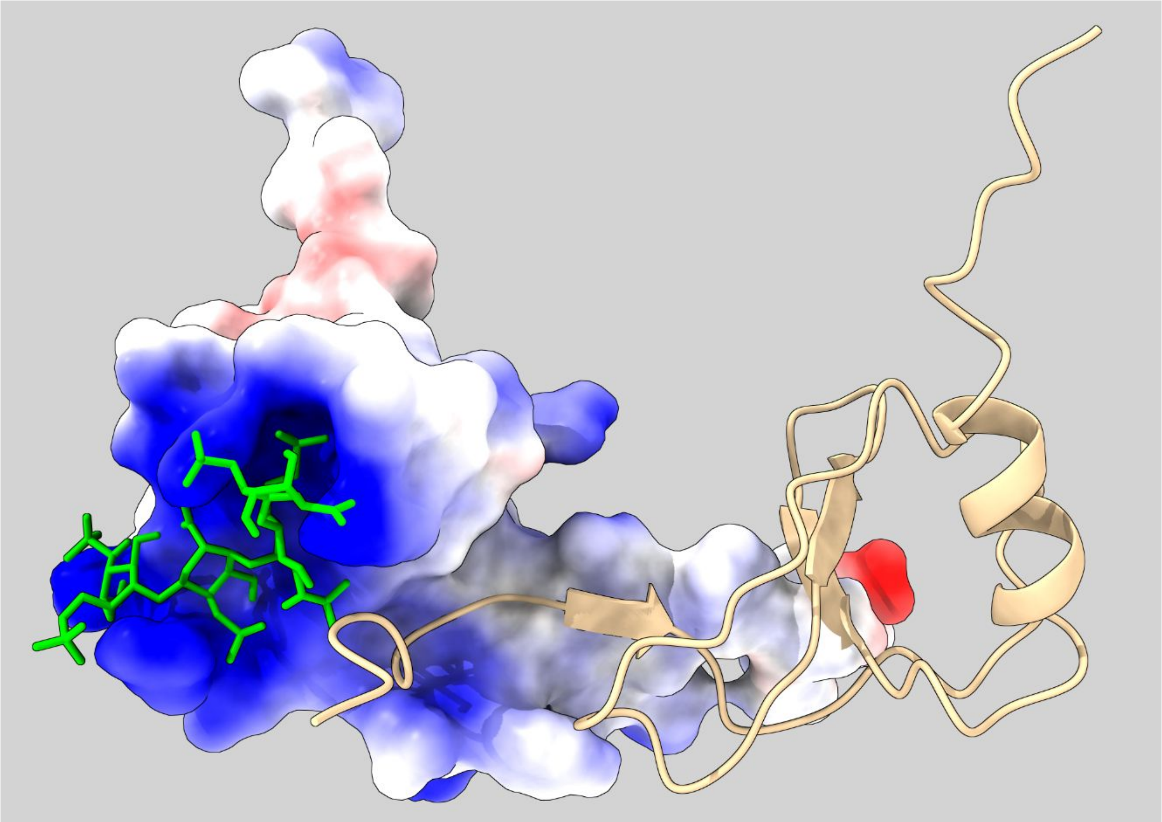

Figure S2. An example structure for the CCL2 dimer:heparin tetrasaccharide complex based on ClusPro calculations.[1–3] The CCL2 monomer interacting with the tetrasaccharide structure was visualized as Coulombic surface, where a gradient of blue-white-red was applied on positive-neutral-negative regions. The second subunit of the dimer is depicted as ribbons for better visibility.

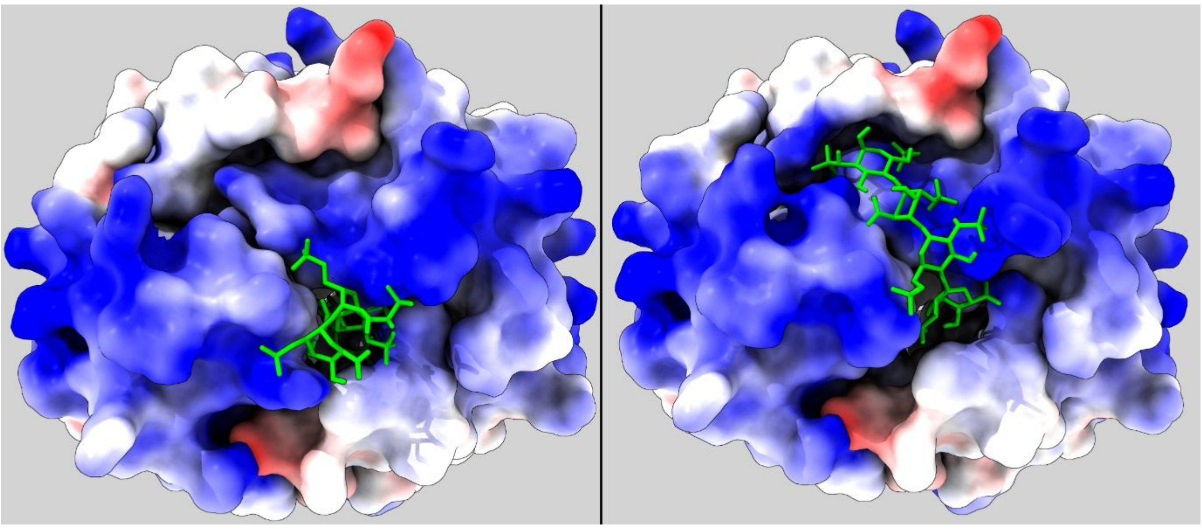

Figure S3. The CXCL4 structure has an extended positive belt around the tetramer. This structure can be stabilized by fondaparinux intercalating into the tetramer (left) or bridging it over from the outside interacting with the positive belt (right). The docking simulations were done by ClusPro with a heparin tetrasaccharide as ligand.[2–4] The CXCL4 tetramer was visualized as Coulombic surface, where a gradient of blue-white-red was applied on positive-neutral-negative regions.

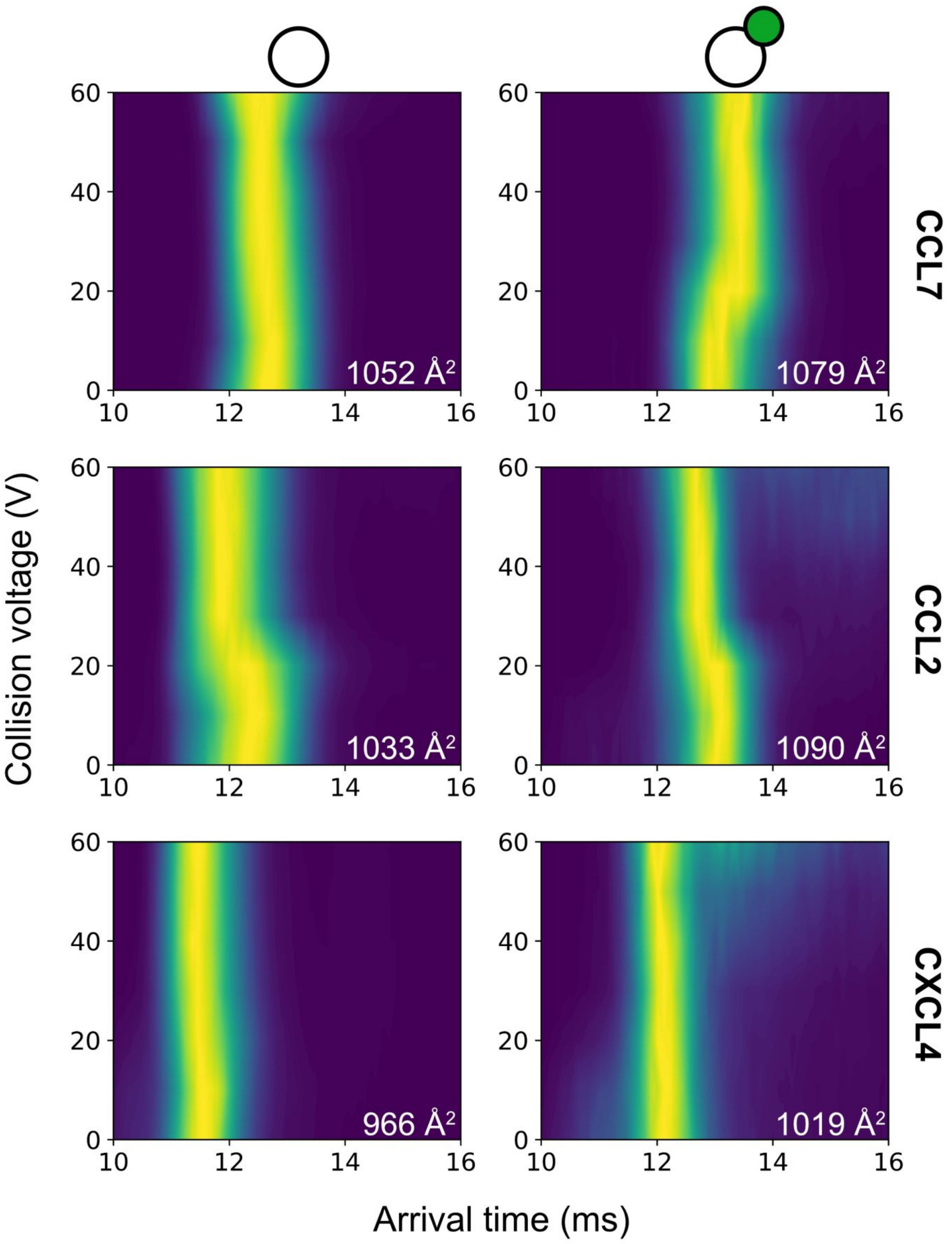

Figure S4. Collision-induced unfolding of the 5+ CCL7, CCL2, and CXCL4 monomers with (right column) and without fondaparinux (left column). The white numbers in the bottom right corner of the panels mark the rotationally-averaged collision cross-section in He drift gas at 0 V collision voltage.

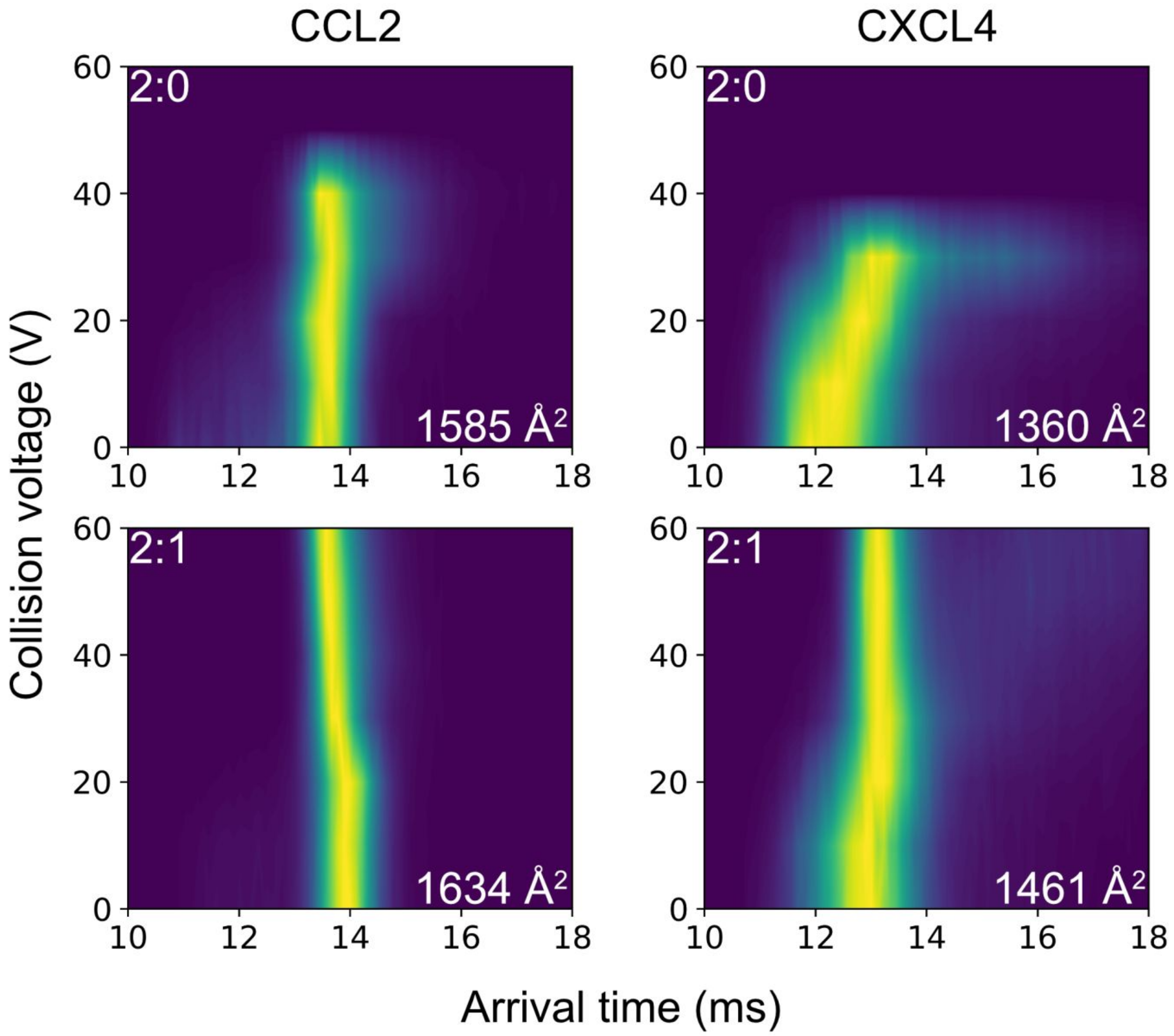

Figure S5. Collision-induced unfolding and dissociation of the 7+ CCL2 and CXCL4 homodimers with (2:1) and without fondaparinux (2:0). The white numbers in the bottom right corner mark the rotationally-averaged collision cross sections in He drift gas.

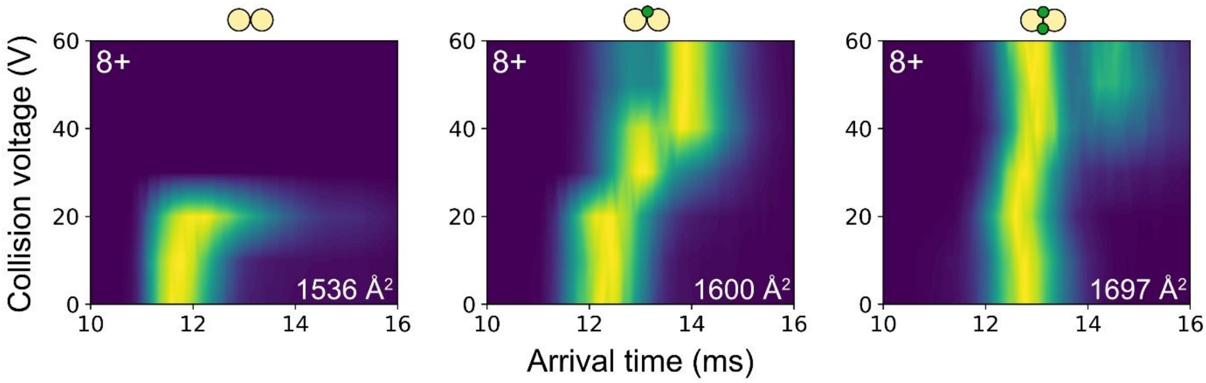

Figure S6. Collision-induced unfolding of the 8+ CCL2 dimer in complex with 0-2 fondaparinux molecules. The white numbers in the bottom right corner mark the rotationally-averaged collision cross sections in He drift gas.

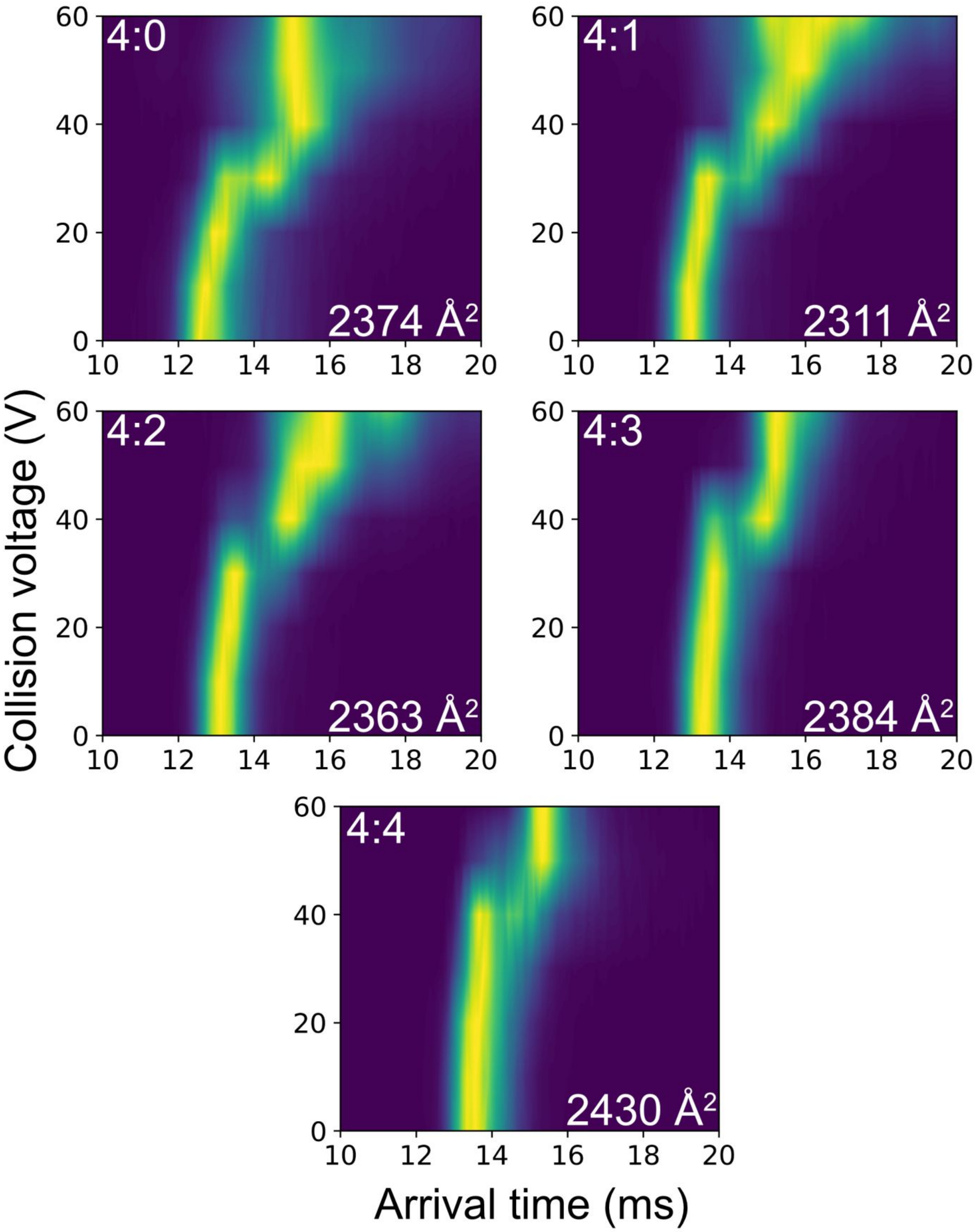

Figure S7: Collision-induced unfolding of the 11+ CXCL4 tetramer in complex with 0-4 fondaparinux molecules. The white numbers in the bottom right corner mark the rotationally-averaged collision cross sections in He drift gas.

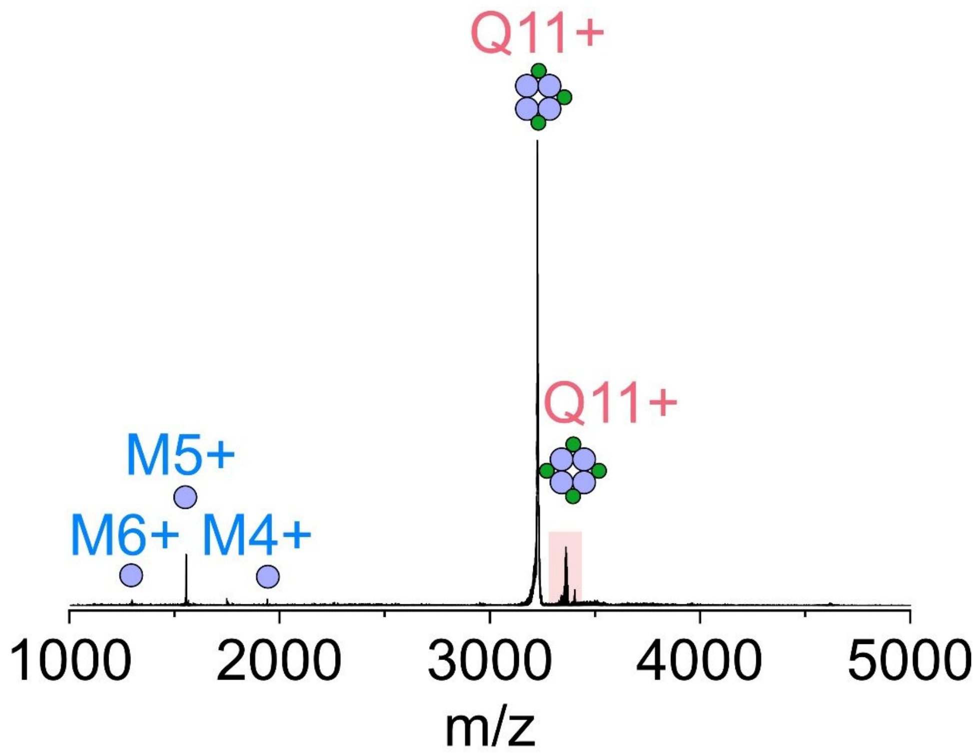

Figure S8. Collision-induced dissociation mass spectrum of the 11+ 4:4 CXCL4:fondaparinux complex at 30 V collision voltage. The parent ion signal is highlighted in red. It is worth noting that the 4:2 complex in Figure 3 is mostly intact at 30 V collision voltage, while the majority of the 4:4 complex dissociates into the 4:3 species.

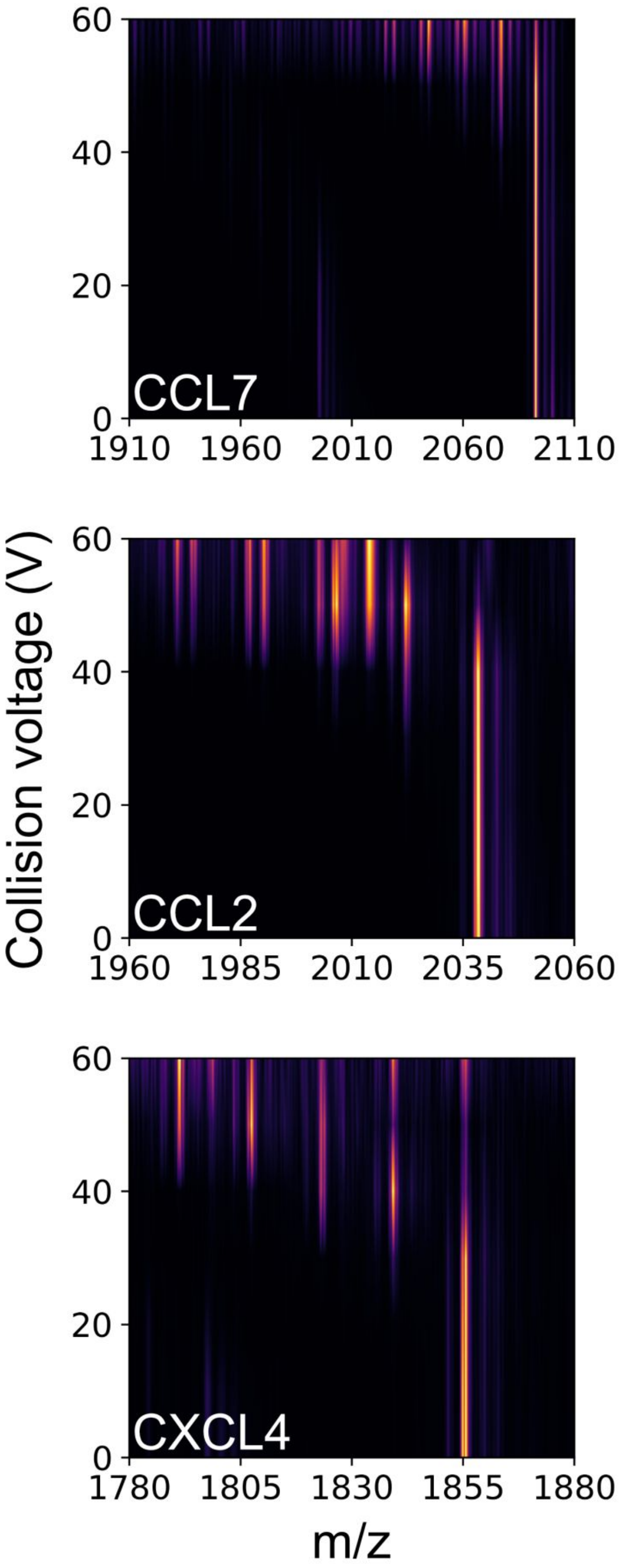

Figure S9: Collision-induced sulfate loss of the 5+ 1:1 chemokine:fondaparinux complexes.

References

(1) Handel, T. M.; Domaille, P. J. Heteronuclear (1H, 13C, 15N) NMR Assignments and Solution Structure of the Monocyte Chemoattractant Protein-1 (MCP-1) Dimer. *Biochemistry* **1996**, *35* (21), 6569–6584. https://doi.org/10.1021/bi9602270.

(2) Meng, E. C.; Goddard, T. D.; Pettersen, E. F.; Couch, G. S.; Pearson, Z. J.; Morris, J. H.; Ferrin, T. E. UCSF ChimeraX: Tools for Structure Building and Analysis. *Protein Science* **2023**, *32* (11), e4792. https://doi.org/10.1002/pro.4792.

(3) Kozakov, D.; Hall, D. R.; Xia, B.; Porter, K. A.; Padhorny, D.; Yueh, C.; Beglov, D.; Vajda, S. The ClusPro Web Server for Protein-Protein Docking. *Nat Protoc* **2017**, *12* (2), 255–278. https://doi.org/10.1038/nprot.2016.169.

(4) Zhang, X.; Chen, L.; Bancroft, D. P.; Lai, C. K.; Maione, T. E. Crystal Structure of Recombinant Human Platelet Factor 4. *Biochemistry* **1994**, *33* (27), 8361–8366. https://doi.org/10.1021/bi00193a025.
